# Keratocystoma of the Parotid Gland: A Systematic Review of Clinicopathologic Characteristics

**DOI:** 10.1002/lio2.70492

**Published:** 2026-07-04

**Authors:** Rijahd Sahmer Boustani, Malte Gronewold, Sönke Maximilian Braß, Efthymios Papazacharias, Michael‐Tobias Neuhaus, Fritjof Lentge, Nils‐Claudius Gellrich, Philipp Jehn, Philippe Korn

**Affiliations:** ^1^ Department of Oral and Maxillofacial Surgery Hannover Medical School Hannover Germany; ^2^ Institute of Pathology, Hannover Medical School Hannover Germany

**Keywords:** histopathology, keratocystoma, parotid gland, salivary gland tumor, systematic review

## Abstract

**Objectives:**

Keratocystoma is a rare benign neoplasm of the parotid gland, histologically characterized by multicystic keratinizing stratified squamous epithelium. Due to its resemblance to squamous malignancies, it represents a diagnostic challenge. This systematic review aims to summarize all published cases of parotid gland keratocystoma and to integrate one newly identified case in order to define its clinicopathologic features and clinical outcomes.

**Methods:**

A systematic literature search of PubMed and Google Scholar was conducted without date restrictions in accordance with PRISMA 2020 guidelines. The protocol was registered in PROSPERO (CRD420251024191). Peer‐reviewed case reports and case series of histopathologically confirmed keratocystoma limited to the parotid gland were included. Demographic, clinical, radiologic, histopathologic, immunohistochemical, therapeutic, and follow‐up data were extracted and descriptively analyzed. Methodological quality was assessed using the Joanna Briggs Institute (JBI) Critical Appraisal Checklists. One additional institutional case treated at Hannover Medical School in March 2025 was included.

**Results:**

Fifteen patients (14 from the literature and one new case) were identified. The mean age was 36.9 years (range: 1–77 years) with a slight male predominance. All tumors presented as painless parotid swellings and were treated surgically, most commonly by partial or lateral parotidectomy. No recurrences were reported during a mean follow‐up of 22.8 months. Histologically, all cases demonstrated consistent squamous epithelial features with immunohistochemical positivity for AE1/AE3, CK5/14, and p63. The newly reported case represents the oldest patient described to date.

**Conclusion:**

Keratocystoma of the parotid gland exhibits reproducible benign clinical behavior and characteristic histopathologic features. Surgical excision appears curative, supporting its classification as a distinct benign salivary gland neoplasm that should be considered in the differential diagnosis of cystic parotid lesions.

## Graphical abstract

Keratocystoma is an exceptionally rare benign salivary gland neoplasm of the parotid gland that may mimic malignant squamous lesions. This systematic review summarizes all published cases and includes one additional institutional case, demonstrating consistent clinicopathologic features, favorable outcomes following surgical excision, and no documented recurrences during available follow‐up. These findings support recognition of keratocystoma as a distinct benign salivary gland entity.

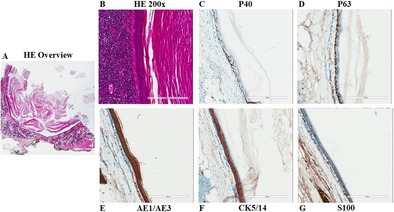

**Abbreviations**
CT: computed tomography
ECD: extracapsular dissection
F: female
FU: follow‐up (months)
IHC: immunohistochemistry
L: left
LP: lateral parotidectomy
M: male
MRI: magnetic resonance imaging
N: no
P: pain
PP: partial parotidectomy
R: recurrence
R: right
SD: symptom duration
SurgTx: surgical treatment
SW: swelling
TP: total parotidectomy
US: ultrasound
Y: yes

## 1 Introduction

Keratocystoma is a rare benign neoplasm of the parotid gland, histologically characterized by multicystic spaces lined with stratified squamous epithelium exhibiting keratinized lamellae [1, 2, 3]. First described in 1999 as a choristoma [4] and later recognized as a distinct entity [1], 14 cases have been reported in the literature to date. This systematic review summarizes all documented cases of keratocystoma of the parotid gland and includes a newly identified case treated at Hannover Medical School in March 2025. To our knowledge, this is the first case of parotid gland keratocystoma in Germany for which both histopathological and immunohistochemical features have been documented in accordance with current diagnostic standards, involving the oldest patient published to date. Clinical presentation, radiologic and histopathologic features, treatment, and outcomes were analyzed across 15 patients. All tumors were managed surgically, with no recurrences reported during follow‐up. This review highlights the consistent benign behavior of keratocystoma and its reproducible histological features. These findings support its recognition as a distinct salivary gland entity that warrants consideration in the differential diagnosis of parotid cystic lesions.

### 1.1 Background

Keratocystomas are benign tumors of the parotid gland, histologically characterized by multicystic structures lined by stratified squamous epithelium. First defined by Nagao et al. in 2002 [1], the lesion has since been variably referred to as choristoma or trichoadenoma [4], creating confusion in classification. Despite its benign nature, the rarity of keratocystoma and its histological resemblance to squamous cell carcinoma, mucoepidermoid carcinoma, and other squamous cell–rich malignancies necessitate thorough differential diagnosis [3, 5]. This review aims to systematically analyze existing literature and integrate new findings from a recently treated case at Hannover Medical School to improve understanding of this rare entity.

## 2 Methods

**FIGURE 1:**
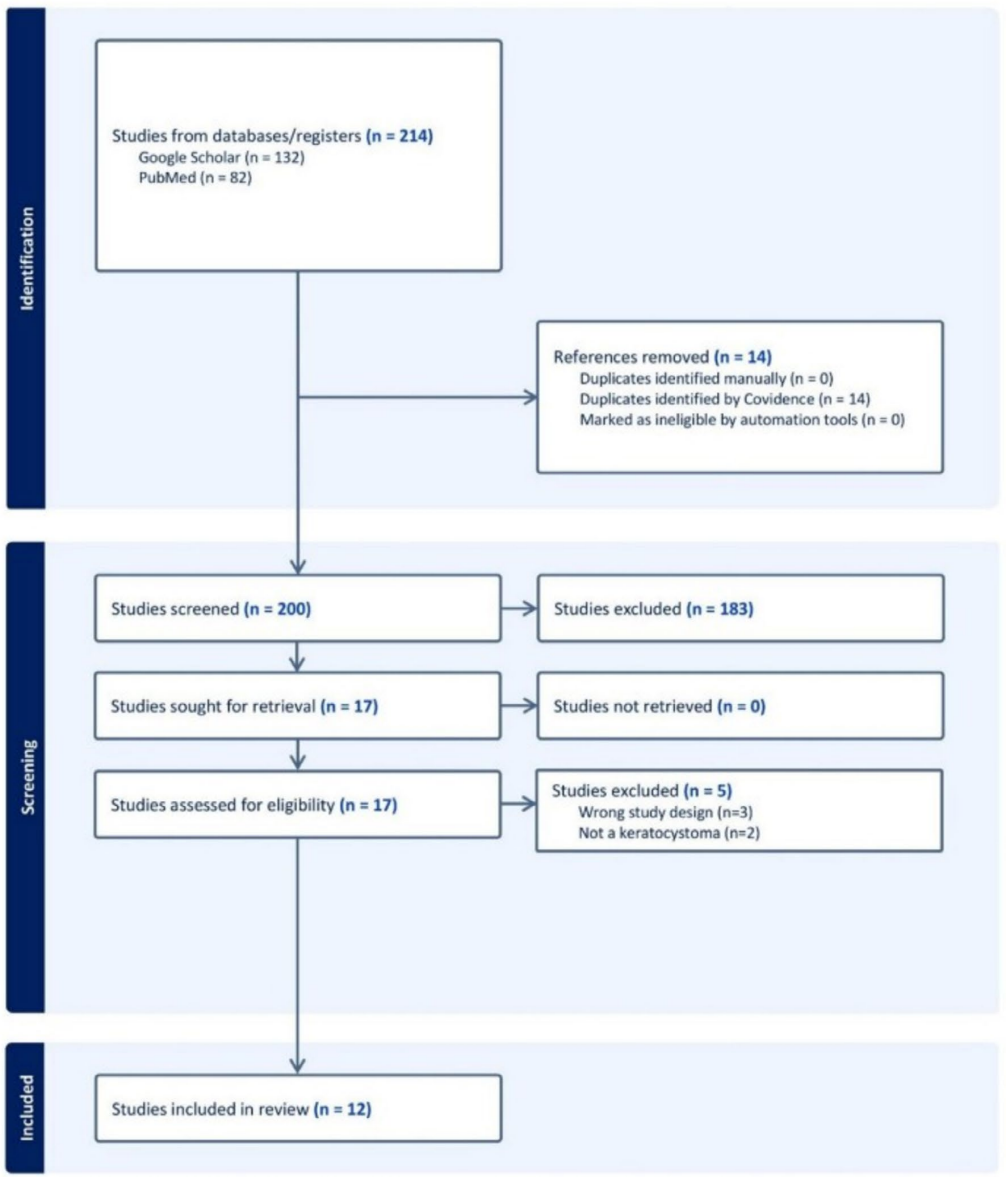
PRISMA flow diagram.

This systematic review was conducted following a registered protocol in PROSPERO (ID: CRD420251024191) and is reported according to PRISMA 2020. A comprehensive literature search was performed in PubMed (MEDLINE) and Google Scholar using combinations of the terms “keratocystoma,” “parotid gland,” “salivary gland,” and “choristoma” (Figure 1). To maximize case identification, the reference lists of all included studies were additionally screened for potentially relevant publications. Only studies published in English were considered. No gray literature was included. Given the rarity of keratocystoma and the limited number of indexed reports, these databases were deemed sufficient to capture all relevant peer‐reviewed cases. Pilot searches in Embase and Web of Science yielded no additional unique results and were therefore not included. The final search was conducted on April 1, 2025, without date restrictions.

Although the term choristoma was initially included in the search to maximize sensitivity, full‐text screening excluded articles using this term when the described lesion did not meet the currently accepted histopathological criteria of keratocystoma, as defined by Nagao et al. and the recent WHO Classification of Head and Neck Tumors [1, 6]. Furthermore, only cases located in the parotid gland were included to ensure anatomical specificity.

### 2.1 Inclusion Criteria

- Peer‐reviewed case reports, case series, or observational studies
- Histopathologically confirmed diagnosis of keratocystoma of the parotid gland
- Sufficient clinical, radiological, histological, and/or immunohistochemical data
- Human subjects of all ages
- Additionally, one unpublished institutional case treated at Hannover Medical School in March 2025 based on identical diagnostic criteria

### 2.2 Exclusion Criteria

- Tumors described outside the parotid gland
- Cases lacking definitive histopathological diagnosis
- Experimental or animal studies
- Reviews, commentaries, conference abstracts, or book chapters

### 2.3 Data Extraction

Titles and abstracts were screened independently by two reviewers using the Covidence platform. Full texts were assessed against eligibility criteria. Data were extracted using a standardized data collection form and recorded in Microsoft Excel. Parameters included demographic details, clinical presentation (e.g., swelling, pain, facial nerve function), imaging studies (ultrasound, CT, MRI), histological features, immunohistochemical profile, treatment modality, recurrence status, and follow‐up duration. In cases of missing data, authors were not contacted.

One newly identified patient treated at Hannover Medical School on March 13, 2025, was included in the dataset. The case fulfilled all inclusion criteria and was fully documented with clinical, surgical, and histological data. Ethical approval for the inclusion of this case was obtained from the local ethics committee at Hannover Medical School (Reference ID: 11858‐BO‐K‐2025). Written informed consent was provided by the legal guardian.

### 2.4 Quality Assessment

The methodological quality of included studies was assessed independently by two reviewers using the Joanna Briggs Institute (JBI) Critical Appraisal Checklists. Case reports were evaluated using the JBI Checklist for Case Reports, whereas studies reporting more than one patient were assessed using the JBI Checklist for Case Series. Any discrepancies were resolved through discussion and consensus.

The assessment focused on the completeness of reporting regarding patient characteristics, clinical presentation, diagnostic work‐up, histopathological findings, treatment, follow‐up, and outcome data. The results of the quality assessment are provided in Table S1.

### 2.5 Statistical Analysis

Due to the rarity of the disease and heterogeneity of the available reports, no meta‐analysis was performed. Descriptive statistics were used to summarize demographic, clinical, radiologic, histopathologic, treatment, and follow‐up characteristics. Continuous variables are presented as means, medians, ranges, and standard deviations where applicable, while categorical variables are reported as frequencies and percentages.

Descriptive statistical analyses were performed using R (Version 4.3.1; R Foundation for Statistical Computing, Vienna, Austria) in a Jupyter Notebook environment. Exploratory statistical tests, including binomial tests, Fisher's exact tests, and Spearman correlation analyses, were performed where appropriate. Given the limited sample size, all statistical findings should be interpreted cautiously.

## 3 Results

A total of 14 published cases of keratocystoma of the parotid gland were identified in the literature, spanning from 2002 to 2024, and supplemented by one newly reported case treated at Hannover Medical School in March 2025. This resulted in a combined cohort of 15 patients included in the present review. All included studies were peer‐reviewed case reports or small case series. The dataset comprises patients from China (5), Japan (3), Italy (2), Germany (2), United States (1), France (1), and United Kingdom (1), reflecting a broad international distribution (Table 1) [1, 2, 3, 5, 7, 8, 9, 10, 11, 12, 13, 14]. Overall, the included studies demonstrated moderate to high reporting quality. The principal limitations identified were incomplete follow‐up reporting and limited methodological detail in conference abstracts and abbreviated reports. The mean patient age was 36.9 years (range: 1–77 years), with a slight male predominance (9 males, 6 females). The median symptom duration prior to diagnosis was 6 months (range: 1–72 months). Swelling was reported in all cases (100%), while pain was noted in 1 case (6.7%). Tumor size ranged from 19 to 40 mm in maximum dimension (Table 2).

**TABLE 1:** Summary of cases following literature research.

Case	Author	Year	Sex	Age	SD	SW	P	Img	SurgTx	R	FU	IHC
1	Nagao et al.	2002	M	18	R	Y	N	—	PP	N	36	AE1/AE3+ CK5/14+; S100−
2	Nagao et al.	2002	M	38	L	Y	N	CT	PP	N	24	AE1/AE3+ CK5/14+; S100−
3	Mei‐Fang Zhang et al.	2010	M	37	L	Y	N	—	PP	N	18	AE1/AE3+ CK5/14+; p63+; p53+; Ki67+
4	Huang et al.	2011	F	29	R	Y	N	—	PP	N	48	AE1/AE3+ CK5/14+; Ki67+; S100−
5	Huang et al.	2012	F	49	R	Y	N	—	PP	N	36	AE1/AE3+; CK5/14+; p63+; S100−
6	Weiya Wang et al.	2015	M	34	L	Y	1	—	PP	N	36	p63+; p53+; Ki67+
7	Komatu et al.	2019	M	18	L	Y	N	CT MRI	LP	N	16	AE1/AE3+ CK5/14+; Ki67+
8	Anparasan et al.	2022	M	75	L	Y	N	MRI	LP	N	15	p63+; p53+; Ki67+
9	Hirata et al.	2014	M	34	L	Y	N	US MRI	LP	N	17	—
10	Saraniti et al.	2022	F	75	L	Y	N	US CT	ECD	N	16	—
11	Klijanienko et al.	2024	M	1	L	Y	N	US MRI	TP	—	—	p53+; Ki67+; p16+
12	Aresta, Girolami et al.	2019	M	51	R	Y	N	US MRI	PP	—	—	AE1/AE3+ CK5/14+; Ki67+
13	Liu et al.	2024	F	2	L	Y	N	US MRI	PP	N	1	p40+; p53+; Ki67+
14	Spahn et al.	2018	F	12	L	Y	N	—	TP	N	10	—
15	Boustani et al.	2025	F	77	L	Y	N	US CT	LP	—	—	AE1/AE3+ CK5/14+; p63+; p40+; S100+/−

**TABLE 2:** Summary of clinical characteristics of reported keratocystoma cases.

Variable	Value/Range
Number of cases	15
Age (mean, range)	36.9 years (1–77 years)
Sex	9 males (60.0%), 6 females (40.0%)
Tumor side	11 left (73.3%), 4 right (26.7%)
Symptom duration (median, range)	6 months (1–72 months)
Tumor size (max. diameter)	19–40 mm
Preoperative facial palsy	1 case (7.0%)
Postoperative facial palsy	4 cases (57.1% of 7 with available data)
Surgical treatment	Partial parotidectomy (*n* = 8; 53.3%) Lateral parotidectomy (*n* = 4; 26.7%) Total parotidectomy (*n* = 2; 13.3%) Extracapsular dissection (*n* = 1; 6.7%)
Recurrence	None observed
Imaging: Ultrasound	6 of 6 patients with available data (100.0%) Missing in 9 of 15 cases
Imaging: CT	4 of 8 patients with available data (50.0%) Missing in 7 of 15 cases
Imaging: MRI	6 of 7 patients with available data (85.7%) Missing in 8 of 15 cases

Radiological imaging was inconsistently reported across cases. Ultrasound (US) was performed in 6 of 6 patients with available data (100%), computed tomography (CT) in 4 of 8 (50%), and magnetic resonance imaging (MRI) in 6 of 7 cases (85.7%). Imaging data were incomplete for several cases, with missing values reported in 9 for ultrasound, 7 for computed tomography, and 8 for magnetic resonance imaging.

Regarding tumor laterality, 11 tumors (73.3%) were located in the left parotid gland and 4 (26.7%) in the right. No statistically significant deviation from a 1:1 distribution was observed (*p* = 0.118; 95% CI: 44.9%–92.2%). Similarly, although the majority of patients were male, the sex distribution was not statistically different from an expected 50:50 ratio (*p* = 0.607; 95% CI: 32.3%–83.7%).

Surgical management primarily consisted of partial parotidectomy, performed in 8 of 15 cases (53.3%). Lateral parotidectomy was employed in 4 cases (26.7%), total parotidectomy in 2 cases (13.3%) and extracapsular dissection in 1 case (6.7%).

Follow‐up data were available for 12 of 15 patients. The mean follow‐up duration was 22.8 months (SD: 13.5), with a median of 17.5 months (range: 1–48 months; IQR: 20.25; CV: 0.59). Follow‐up information was missing in 3 cases.

No recurrences were reported among patients with available follow‐up data.

Preoperative facial nerve weakness was observed in 1 of 11 patients (9.1%) with available data, while information was missing in 4 of 15 cases. Postoperative facial nerve dysfunction occurred in 4 of 7 patients (57.1%) with available data. In 8 of 15 cases (53.3%), no postoperative facial nerve status was documented. A Fisher's exact test revealed no significant association between surgical technique and facial nerve dysfunction (*p* = 0.485).

A correlation analysis between symptom duration and tumor volume showed no significant association (*p =* 0.525). A statistical summary of key variables including *p‐*values is presented in Table 3.

**TABLE 3:** Statistical summary table with *p*‐values.

Variable	Categories/Values	Valid (%)	Missing	Test	*p*
Sex	M (60.0%), F (40.0%)	15 (100.0%)	0	Binomial test	0.607
Side	L (73.3%), R (26.7%)	15 (100.0%)	0	Binomial test	0.118
Pain	Y (6.7%), N (93.3%)	15 (100.0%)	0	Binomial test	0.001
Preoperative Facial Palsy	Y (9.1%), N (90.9%)	11 (73.3%)	4	Binomial test	0.006
Postoperative Facial Palsy	Y (57.1%), N (42.9%)	7 (46.7%)	8	Binomial test	0.625
Swelling	Y (100.0%)	15 (100.0%)	0	—	—
Recurrence	N (100.0%)	12 (80.0%)	3	—	—
Ultrasound	Y (100.0%)	6 (40.0%)	9	Binomial test	0.146
CT	Y (50.0%), N (50.0%)	8 (53.3%)	7	Binomial test	1.000
MRI	Y (85.7%), N (14.3%)	7 (46.7%)	8	Binomial test	0.219

Immunohistochemical profiles were reported in 12 of 15 cases. Among those, consistent staining patterns were observed, with positivity for cytokeratins AE1/AE3 and CK5/6/14, and p63, while negative for S100 and mostly positive for Ki67 in low expression.

## 4 Discussion

Keratocystoma of the parotid gland is a rare benign tumor entity that remains largely unfamiliar to many clinicians and pathologists due to its rarity. It was initially described by Seifert et al. in 1999 as a choristoma based on the presence of ectopic structures such as keratinizing squamous epithelium, sebaceous glands, and cartilage [4]. However, this interpretation was later challenged by Nagao et al., emphasizing that the tumor originates from the epithelial parenchyma of the parotid gland rather than representing a developmental malformation [1]. This subtle but important distinction is rooted in histopathological criteria: whereas choristomas consist of normal tissue in an ectopic location [15], keratocystomas exhibit a cystic epithelial proliferation within the anatomical boundaries of the parotid gland, lacking features of heterotopia [1].

Nevertheless, the 5th Edition of the World Health Organization Classification of Head and Neck Tumors added keratocystoma in 2022 as a very rare benign tumor entity of the parotid gland [6, 16]. Notably, the case involving a 77‐year‐old patient constitutes the oldest reported instance of keratocystoma in the parotid gland documented in the literature to date. This finding contrasts with earlier assumptions by Nagao et al., who suggested that keratocystoma predominantly affects younger individuals [1]. The observed mean age challenges previously reported assumptions and indicates that keratocystoma can occur across a broader age spectrum than suggested by Wang et al. [12], who limited the affected age spectrum to between 8 and 49 years.

**FIGURE 2:**
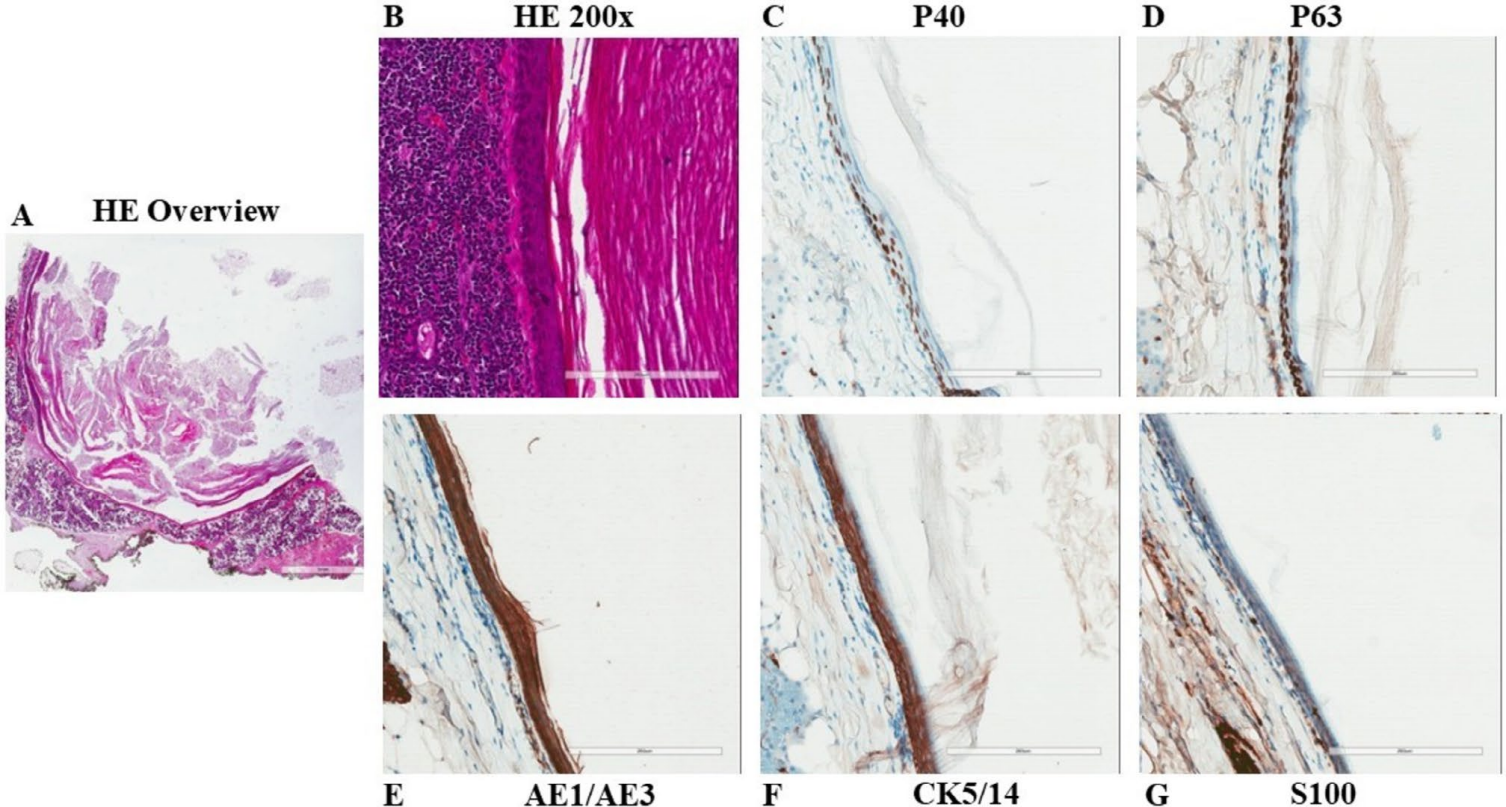
Histopathological and immunohistochemical staining.

Histologically, all tumors showed consistent patterns: multicystic architecture lined by keratinizing stratified squamous epithelium, often filled with lamellar keratin and occasionally accompanied by squamous nests. Importantly, glandular, myoepithelial, or dermoid elements were absent [1, 3, 5]. The immunohistochemical profile observed across reported cases of keratocystoma demonstrates consistent expression of basal and squamous epithelial markers. AE1/AE3, a broad‐spectrum cytokeratin, confirms the epithelial origin of the tumor. CK5/6/14, representing high‐molecular‐weight cytokeratins typically expressed in basal and squamous epithelium, further supports squamous differentiation. The nuclear marker p63, indicative of basal or myoepithelial lineage, is frequently positive in squamous epithelium and benign epithelial proliferations. In contrast, S100 is typically negative in keratocystoma, which helps to distinguish it from neoplasms of myoepithelial or neural origin. Ki‐67, a proliferation marker, shows only low expression, which underscores the benign biological behavior of this tumor entity [2, 3]. In the case treated at Hannover Medical School, immunohistochemical analysis revealed expected positive staining for AE1/AE3, CK5/14, p40, and p63. S100 showed weak and nonspecific staining (+/−) (Figure 2C–G). Hematoxylin and eosin (HE) staining demonstrated the typical multicystic architecture and squamous epithelial lining (Figure 2A,B). These findings are consistent across reported cases and support the classification of keratocystoma as a distinct benign squamous epithelial neoplasm.

The clinical differential diagnosis poses a significant challenge. Keratocystoma may mimic pleomorphic adenomas, Warthin tumors, as well as squamous cell carcinoma or mucoepidermoid carcinomas, which all occur by mostly painless swelling. However, the absence of cytological atypia, low proliferative activity (e.g., Ki‐67 limited to the basal layer), and the lack of perineural or lymphovascular invasion argue against malignancy [1, 5]. Moreover, the absence of mucous‐producing cells or mucicarmine staining helps distinguish keratocystoma from mucoepidermoid carcinoma. In addition, the absence of preoperative facial nerve palsy may serve as a supportive clinical indicator of benign behavior. Only one out of fifteen patients (6.7%) exhibited facial nerve dysfunction at the time of diagnosis in the reviewed cohort. While this finding cannot reliably differentiate keratocystoma from other benign entities such as pleomorphic adenoma, it may assist in distinguishing it from malignant tumors, where facial nerve involvement is more frequently observed and a predictor for a worse overall and disease‐free survival [17].

A correlation analysis between symptom duration and tumor volume showed no significant association (*p* = 0.525), suggesting that larger tumors were not necessarily associated with longer clinical latency. This clinical course resembles that of other benign parotid gland tumors, such as pleomorphic adenoma, which typically present as slow‐growing, painless masses that displace rather than infiltrate adjacent structures, including the facial nerve [18].

Surgical management in all reported cases consisted primarily of partial parotidectomy. No recurrences were documented during follow‐up. The reported follow‐up durations ranged from 1 to 48 months, with a median of 17.5 months and a mean follow‐up time of 22.8 months.

(SD: 13.5; IQR: 20.25; CV: 0.59). Postoperative facial nerve dysfunction occurred in 4 of 7 patients with available data (57.1%). This corresponds to 21.0% of the total cohort, assuming no dysfunction in cases with missing data. While this rate appears comparable to that observed in other benign parotid tumors, including pleomorphic adenomas [1, 5], interpretation is limited by the high proportion of missing data (8 of 15 cases). Therefore, no definitive conclusions can be drawn regarding the overall incidence of postoperative facial nerve impairment in keratocystoma. In our series, no statistically significant link between surgical technique and nerve impairment was found.

The newly reported case from Hannover Medical School involved a painless, 36 mm lesion of the parotid gland, which was surgically treated via lateral parotidectomy. This approach is commonly employed for benign tumors located within the lateral lobe of the parotid gland and offers reliable exposure while preserving the facial nerve [19, 20]. Notably, in one case included in our review, extracapsular dissection (ECD) was utilized as the surgical technique. ECD is considered a less invasive alternative to traditional parotidectomy, aiming to preserve facial nerve function by maintaining a safe margin of 2 to 3 mm from uninvolved parotid tissue. ECD may represent a suitable surgical option for select benign parotid lesions, particularly those located peripherally and without involvement of the deep lobe. However, current data on its long‐term oncologic safety and recurrence rates remain limited [21, 22, 23, 24, 25, 26].

### 4.1 Limitations

The rarity of keratocystoma makes it difficult to draw broad conclusions. The available data are limited to single case reports or small series, often with incomplete documentation or follow‐up. Furthermore, follow‐up duration varied considerably across reports and was as short as 1 month in some cases, limiting assessment of long‐term recurrence rates and postoperative facial nerve outcomes.

The absence of uniform diagnostic and reporting standards further complicates interpretation. A limitation of this review is the use of only two electronic databases; however, pilot searches in Embase and Web of Science confirmed no additional yield beyond PubMed and Google Scholar, making this approach appropriate for such an ultra‐rare entity. Despite these constraints, the present review provides the most comprehensive synthesis of this lesion to date and serves as a reference point for future documentation and classification.

## 5 Conclusion

Keratocystoma of the parotid gland is a rare but well‐defined benign tumor with consistent clinical and histopathological features. Surgical resection remains curative, with excellent prognosis and no documented recurrences. The newly reported case from Hannover Medical School represents the oldest patient with keratocystoma described to date (77 years), thereby expanding the known age spectrum of this tumor entity. Unlike pleomorphic adenomas, which are prone to recurrence, keratocystomas have not demonstrated such behavior in the literature.

These observations reinforce the recognition of keratocystoma as a distinct salivary gland neoplasm and underline the need for prospective data collection and more clearly defined diagnostic criteria in future studies.

## Funding

The authors have nothing to report.

## Ethics Statement

The study was approved by the local ethics committee at Hannover Medical School, Germany (approval ID: 11858‐BO‐K‐2025). It followed all applicable standards for good scientific practice and the Declaration of Helsinki.

## Conflicts of Interest

The authors declare no conflicts of interest.

## Supporting information


**Table S1:** Methodological quality assessment of included studies using the Joanna Briggs Institute (JBI) critical appraisal checklists.

## Data Availability

Data sharing not applicable to this article as no datasets were generated or analyzed during the current study.
