# Supplementary material for: Keratocystoma of the Parotid Gland: A Systematic Review of Clinicopathologic Characteristics
Source: Laryngoscope Investig Otolaryngol. 2026 Jul 4;11(4):e70492. doi: 10.1002/lio2.70492 (PMC13332516; doi:10.1002/lio2.70492)
Supplement: Supplementary file 1 — Table S1: Methodological quality assessment of included studies using the Joanna Briggs Institute (JBI) critical appraisal checklists. [file LIO2-11-e70492-s001.pdf]

Supplementary Table S1

Methodological quality assessment of included studies using the Joanna Briggs Institute (JBI) Critical Appraisal Checklists

Y = yes; N = no; U = unclear; NA = not applicable. Score indicates the number of "yes" responses among applicable checklist items.
Case reports were assessed using the JBI Checklist for Case Reports (Q1-Q8). Case series were assessed using the JBI Checklist for Case Series (Q1-Q10).

Study	Design	Checklist	Q1	Q2	Q3	Q4	Q5	Q6	Q7	Q8	Q9	Q10	Score
Nagao et al., 2002	Case series	JBI Case Series	Y	Y	Y	U	U	Y	Y	Y	Y	NA	7/9
Huang et al., 2012	Case series	JBI Case Series	Y	Y	Y	U	U	Y	Y	Y	Y	NA	7/9
Zhang et al., 2010	Case report	JBI Case Report	Y	Y	Y	Y	Y	Y	U	Y	NA	NA	7/8
Wang et al., 2015	Case report	JBI Case Report	Y	Y	Y	Y	Y	Y	U	Y	NA	NA	7/8
Hirata et al., 2014	Case report	JBI Case Report	Y	Y	Y	Y	Y	Y	Y	Y	NA	NA	8/8
Komatsu et al., 2020	Case report	JBI Case Report	Y	Y	Y	Y	Y	Y	Y	Y	NA	NA	8/8
Aresta et al., 2019	Case report	JBI Case Report	Y	Y	Y	Y	Y	U	U	Y	NA	NA	6/8
Saraniti et al., 2022	Case report	JBI Case Report	Y	Y	Y	Y	Y	Y	Y	Y	NA	NA	8/8
Anparasan et al., 2022	Case report	JBI Case Report	Y	Y	Y	Y	Y	Y	Y	Y	NA	NA	8/8

Liu et al., 2024	Case report	JBI Case Report	Y	Y	Y	Y	Y	Y	Y	Y	NA	NA	8/8
Klijanienko et al., 2025	Case report	JBI Case Report	Y	U	Y	Y	Y	Y	U	Y	NA	NA	6/8
Spahn et al., 2018	Conference abstract	JBI Case Report	Y	U	Y	U	Y	Y	Y	Y	NA	NA	6/8

Checklist items

JBI Checklist for Case Reports (Q1-Q8)

Q1. Were patient demographic characteristics clearly described?
Q2. Was the patient history clearly described and presented as a timeline?
Q3. Was the current clinical condition of the patient on presentation clearly described?
Q4. Were diagnostic tests or assessment methods and results clearly described?
Q5. Was the intervention or treatment procedure clearly described?
Q6. Was the post-intervention clinical condition clearly described?
Q7. Were adverse events or unanticipated events identified and described?
Q8. Does the case report provide takeaway lessons?

JBI Checklist for Case Series (Q1-Q10)

Q1. Were there clear criteria for inclusion in the case series?
Q2. Was the condition measured in a standard and reliable way for all participants?
Q3. Were valid methods used for identification of the condition for all participants?
Q4. Was the case series consecutive inclusion?
Q5. Was the case series complete inclusion?
Q6. Was there clear reporting of participant demographics?
Q7. Was there clear reporting of clinical information?
Q8. Were outcomes or follow-up results clearly reported?
Q9. Was there clear reporting of the presenting site or relevant clinical demographic information?
Q10. Was statistical analysis appropriate?
